# Tense Ascites in Cirrhosis: Paracentesis With Albumin Infusion Versus Spontaneous Ascites Filtration

**DOI:** 10.1155/1994/94354

**Published:** 1994

**Authors:** Simon C. Robson

**Affiliations:** MRC UCT Liver Centre Groote Schuur Hospital Cape Town South Africa


simple Pringle manoeuver, the most common situation, or less frequently complete hepatic vascular exclusion by clamping the hepatic pedicle and the vena cava below and above the liver when the tumor is massive and/or badly located, i.e., close to the hepatic veins or inferior vena cava.

Associated aorta clamping does not offer any further advantage and is associated with its own complications. For this reason, it appears unnecessary in our practice today.

### REFERENCES

1. Delva, E., Camus, Y., Nordlinger, B., Hannoun, L., Parc, R., Deriaz, H., Lienhart, A. and Huguet, C. (1989) Vascular occlusion for liver resections. Operative management and tolerance to hepatic ischemia: 142 cases. *Ann Surg.*, **209**, 211–128
2. Nagasue, N., Yukaya H., Ogawa, Y., Hirose, S. and Okita, M. (1985) Segmental and subsegmental resections of the cirrhotic liver under hepatic inflow and outflow occlusion. *Br. J. Surg.*, **72**, 565–568
3. Bismuth, H., Castaing, D. and Garden, O. J. (1989) Major hepatic resection under total vascular exclusion. *Ann Surg.*, **210**, 13–19
4. Heaney, J. P., Stanton, W. K., Halbert, D. S., Seidel, J. and Vice, T. (1966) An improved technique for vascular isolation to the liver experimental study and case reports. *Ann Surg.*, **163**, 237–241
5. Huguet, C., Addario-Chieco, P., Gavelli, A., Arrigo, E., Harb, J. and Roger-Clement, R. (1992) Technique of hepatic vascular exclusion for extensive liver resection. *Am. J. Surg.*, **163**, 602–605.
6. Huguet, C., Nordlinger, B., Galopin, J. J., Bloch, P. and Gallot, D. (1987) Normothermic hepatic vascular exclusion for extensive hepatectomy. *Int. Surg.*, **72**, 78–81
7. Huguet, C., Gavelli, A., Addario-Chieco, P., Bona, S., Harb, J., Joseph, J. M., Jobard, J., Gramaglia, M. and Lasserre, M. (1992) Liver ischemia for hepatic resection: Where is the limit? *Surgery*, **111**, 251–259

Prof. Claude Huguet
Centre Hospitalier Princesse Grace
BP No 480
MC 98082 Monaco Cedex
Monaco

### TENSE ASCITES IN CIRRHOSIS: PARACENTESIS WITH ALBUMIN INFUSION VERSUS SPONTANEOUS ASCITES FILTRATION

### ABSTRACT

*Bruno, S., Borzio, M., Romagnoni, M., Battezzati, P. M., Rossi, S., Chiesa, A., Podda, M. (1992) Comparison of spontaneous ascites filtration and reinfusion with total paracentesis with intravenous albumin infusion in cirrhotic patients with tense ascites. BMJ*; **304**: 1655–1658.

***Objective*—To compare the effectiveness and safety of spontaneous ascites filtration and reinfusion and total paracentesis plus intravenous albumin infusion in cirrhotic patients with tense ascites.**

***Design*—Randomised trial of the two treatments.**

***Setting*—Teaching hospital and district general hospital in Milan.**

***Patients*—45 consecutive cirrhotic patients with recurrent tense ascites and urinary sodium excretion rate < 20 mmol/day. 35 fulfilled admission criteria and completed the study. 17 received spontaneous ascites filtration and 18 paracentesis plus albumin infusion.**

***Main outcome measures*—Body weight; urinary volume; serum and urinary electrolyte, serum fibrinogen, and plasma aldosterone concentrations; and plasma renin activity before the procedure and 24 hours and eight days afterwards.**

***Results*—Both procedures were effective in all patients. Weight decreased in both groups and showed no substantial increase after eight days. In patients receiving ascites filtration, values decreased significantly ($p < 0.01$) after 24 hours for platelet count (mean relative change 0.92; 99% confidence interval 0.86 to 0.98) and serum fibrinogen concentration (0.92; 0.88 to 0.98) but returned to pretreatment values after eight days; no laboratory and clinical signs of disseminated intravascular coagulation were noted. Three patients in this group had fever, which receded spontaneously. One patient in each group had dilutional hyponatraemia.**

***Conclusions*—Spontaneous ascites filtration and reinfusion is an effective treatment for tense ascites. Reinfusion of the patients's concentrated proteins provides savings without compromising safety.**

### PAPER DISCUSSION

KEY WORDS: Ascites, cirrhosis, paracentesis, ascites filtration.

This paper reports on an randomised and controlled evaluation of a gravity dependent ascites filtration, concentration and reinfusion device originally described by Landini *et al.*[1] Thirty five of 45 consecutive patients with cirrhosis (predominantly alcohol related; Child's score B) complicated by tense ascites and decreased urinary sodium excretion rates were randomly assigned to either spontaneous ascites filtration and reinfusion or to total paracentesis plus intravenous albumin infusion. All diuretic therapy was discontinued for the duration of the study, patients were kept in hospital, intake of sodium limited to 20 mmol/day and fluid restricted to 500 mL if serum sodium levels were less than 130 mmol/L or otherwise to 1L/day. Patients were assessed at 24 hours and 8 days following these interventions and the outcome suggested that both procedures were equally effective and safe. The authors conclude that reinfusion of concentrated ascites proteins is less expensive than standard albumin infusions and does not compromise patient safety.

Ascites is the most common complication of cirrhosis and is associated with about 50% of deaths in these patients[2]. Cirrhotic ascites carries a mortality rate of up to 40% in the first year and the prognosis is critically dependent on the progress and severity of the underlying liver disease[3–5]. The only therapy which can significantly improve patient survival remains liver transplantation[6] and it is important to consider the high morbidity and mortality rates associated with the aggressive management of ascites in patients with end-stage liver disease. In this setting for example, peritoneovenous shunts have been shown to be hazardous and ineffective[7].

The majority of patients can be effectively treated with standard medical interventions which comprise of dietary salt restriction and diuretic agents of which spironolactone 150–300 mg/day is the initial drug of choice[8]. Unfortunately, a substantial proportion of cirrhotic patients develop refractory ascites usually in the setting of progressive liver disease. Procedures such as large volume and more recently, single-volume total paracentesis have been advocated by several groups and demonstrated to be rapid, safe and effective. Paracentesis, one of the oldest modalities of treatment for ascites, was largely superseded by diuretic therapy as a result of the perception that this intervention was hazardous and associated with the development of hypovolaemia and electrolyte disturbances[7,8]. The concurrent intravenous administration of albumin, stabilised human serum or plasma preparations are commonly recommended in an attempt to maintain intravascular volume, reducing the incidence of renal impairment and hyponatraemia and thereby promoting earlier discharge from hospital[4,5,9]. The real financial impact of this "cost saving measure" may however be lessened by increased rates of readmission related to the return of ascites, increased physician time commitments and the potential expenditure associated with the implicit difficulties related to the use of human plasma derivatives. The use of less expensive colloid such as haemaccel or dextran as albumin substitutes appears to be less satisfactory and may be associated with more immediate adverse effects[10].

The prior enthusiasism for peritoneovenous shunting has been tempered by the failure of any form of shunt to prolong life and the many complications to the procedure in patients with severe ascites including disseminated intravascular coagulopathy and sepsis[7,11]. Attempts to safely recirculate concentrated endogenous ascites have been documented over the last few decades. Conservation of plasma proteins, opsonic activity and complement would be of further potential advantage in that the risk of infection and spontaneous bacterial peritonitis may be decreased as in the instance of diuretic therapy[12]. However, the concurrent intravenous infusion of injurious or inflammatory mediators, as for example cytokines, activated complement proteins and procoagulants or the inadvertent administration of endotoxins or bacteria in the concentrated ascites protein preparations may result in significant morbidity and even death. The once popular Rhodiascit device is now considered obsolete mainly because of these concerns and the tendency of patients to develop coagulation abnormalities following readministration of the concentrated ascites protein and the time constraints associated with its use[7,13]. The concentration and reinfusion of ascites back into the abdominal cavity may be a safer option in many instances.

The description of the further clinical application of a filtration and concentration device technologically adapted for bedside use is therefore timely and predictable, given the increasing number of published studies concerned with the benefits of and indications for paracentesis. Despite the obvious benefits of liver transplantation, many patients with end-stage liver disease are not considered suitable candidates and must rely on medical modalities of treatment. Patients awaiting transplantation require safe and effective palliative therapies for refractory ascites that should not compromise their chances of survival and of receiving a graft. The procedure of spontaneous ascites filtration and reinfusion described appears easy to perform but usually took over twice the time of a total paracentesis and albumin infusion and up to 10 hours in one case. The volume of ascites removed was similar and of the order of 6 to 7 L in both instances. The therapeutic benefits of total paracentesis followed by the maintainance of intravascular volume were comparable in both groups up to 8 days in the absence of additional diuretic treatment. The observed decreases in platelet counts and fibrinogen levels following ascites reinfusion are in keeping with a mild consumptive coagulopathy. Unfortunately the methods whereby fibrinogen and fibrin degradation product measurements are obtained are not described. Fibrinogen concentrations in serum (sic) are reported and despite assurances that "no laboratory and clinical signs of disseminated intravascular coagulation were noted", the data to substantiate this conclusion[14,15] is not given. It is unclear from the reported methodology whether heparin was administered intraperitoneally to prevent fibrin clot formation within the haemofilter[1,7]. Fever is alternatively described in three (abstract) or two patients (results and discussion) following reinfusion of ascites protein concentrates but these events were apparently self-limiting and did not require specific intervention. Whether these events were associated with endotoxaemia or bacteraemias is not reported.

The comparable results obtained in this initial study for both modalities of treatment suggest that at least in the short term, reinfusion of ascitic protein concentrates can be relatively safe if the procedure is confined to patients with reasonable parameters of haemostasis and renal function with no evidence for spontaneous bacterial peritonitis. The utilisation of diuretics following paracentesis and reinfusion of ascitic protein concentrates would make clinical sense and could significantly delay the reaccumulation of ascites and the need for repeat paracentesis as this procedure does not improve the underlying abnormalities in systemic and renal haemodynamics or sodium retention[7]. Certainly the repeated application of spontaneous ascites filtration and reinfusion may result in complications similar to those experienced in the setting of chronic peritoneovenous shunting with a clinically significant consumptive coagulopathy once homeostatic mechanisms already compromised by liver synthetic dysfunction are overwhelmed[11,14]. The widespread re-introduction of ascites reinfusion therefore seems premature and is limited by Catch 22 scenarios in more ways than one.

### REFERENCES

1. Landini, S., Coli, U., Fracasso, A., Morachiello, P., Righetto, F., Scanferia, F. *et al.* (1985) Spontaneous ascites filtration and reinfusion (SAFR) in cirrhotic patients. *Int. J. Artif. Organs*, **8**, 227–280
2. Trey, C. and Trey, G. (1990) Complications of cirrhosis: ascites and hepatic encephalopathy. *Current Opinion in Gastorenterology*, **6**, 365–369
3. Stanley, M. M., Ochi, S., Lee, K. K. *et al.* (1989) The Veterans Administration Co-operative Study on treatment of alcoholic cirrhosis with ascites. Peritoneovenous shunting as compared with medical treatment in patients with alcoholic cirrhosis and massive ascites. *N. Engl. J. Med.*, **321**, 1632–1638
4. Quintero, E., Arroyo, V., Bory, F. *et al.* (1985) Paracentesis versus diuretics in the treatment of cirrhotics with tense ascites. *Lancet*, **i**, 611–613

5. Gines, P., Tito, L., Arroyo, V. *et al.* (1988) Randomized comparative study of therapeutic paracentesis with and without intravenous albumin in cirrhosis. *Gastroenterology*, **94**, 1493–1502
6. Maddrey, W. C. and van Thiel, D. H. (1988) Liver Transplantation: an overview. *Hepatology*, **8**, 948–959
7. Arroyo, V., Gines, P., Jimenez, W. and Rodes, J. (1991) Ascites, renal failure, and electrolyte disorders in cirrhosis. Pathogenesis, diagnosis, and treatment. *Oxford Textbook of Clinical Hepatology*, **1**:(**9**), 427–470. Edited by Neil McIntyre, Jean-Pierre Benhamou, Johannes Bircher, Mario Rizzetto, Juan Rodes, Oxford University Press
8. Veldhuysen van Zanten, S. J. O. and Hunt, R. H. (1991) Randomized controlled trials of the medical treatment of cirrhotic ascites. *Eur. J. of Gastroent. Hepatology*, **3**, 351–359
9. Veldhuysen van Zanten, S. J. O. (1988) More on the comparison of paracentesis versus diuretics in the treatment of patients with tense ascites. *Gastroenterology*, **95**, 257–258
10. Planas, R., Gines, P., Arroyo, V., Llach, J., Panes, J., Vargas, V. *et al.* (1990) Dextran-70 versus albumin as plasma expanders in cirrhotic patients with tense ascites treated with total paracentesis. Results of a randomized study. *Gastroenterology*, **99**, 1736–1744
11. Conn, H. O. (1989) Workshop of peritoneovenous shunts in the management of ascites. *Trans. Am. Soc. Artif. Intern. Organs*, **35**, 160–177
12. Runyon, B. A., Antillon, M. R. and Montano, A. A. (1989) Effect of diuresis versus therapeutic paracentesis on ascitic fluid opsonic activity and serum complement. *Gastroenterology*, **97**, 158–162
13. Wilkinson, S. P., Henderson, J. and Davidson, A. R. (1975) Ascites reinfusion using the Rhodiascit apparatus—clinical experience and coagulation abnormalities. *Postgrad Med. J.*, **51**, 583–587
14. Carr, J. M. (1989) Disseminated intravascular coagulation in cirrhosis. *Hepatology*, **10**, 103–110
15. Kruskal, J. B., Robson, S. C., Franks, J. and Kirsch, R. E. (1992) Elevated fibrin and fibrinogen related antigens in patients with liver disease. *Hepatology*, **16**, 920–923

Simon C. Robson
MRC UCT Liver Centre
Groote Schuur Hospital
Cape Town
South Africa

